# Simultaneous Label-free Imaging of Nucleolar Dynamics and Subcellular Metabolic Shifts Across Tissue Contexts

**DOI:** 10.1101/2025.10.07.681013

**Published:** 2025-10-08

**Authors:** Kunzan Liu, Ellen L. Kan, Honghao Cao, Jiashu Han, Giovanni de Nola, Linda G. Griffith, Eliezer Calo, Sixian You

## Abstract

The nucleolus is essential for ribosome biogenesis and for regulating cellular responses to growth and stress, and its relationship to cellular metabolic activity and functional state highlights its potential as a biomarker of cellular health. However, challenges in contrast multiplexing and high-resolution isotropic three-dimensional (3D) imaging hinder the non-invasive, simultaneous assessment of nucleolar activity and subcellular metabolic maps across different tissue contexts, especially in complex 3D environments. To fully harness the nucleolus’s potential as a biomarker and diagnostic target, we present a multimodal imaging platform that combines third harmonic generation (THG) imaging with metabolic autofluorescence of NAD(P)H and FAD to study structural and metabolic nucleolar dynamics. Enabled by a high-power multimode fiber source and an axial deblurring network, we achieved ∼ 400 nm isotropic resolution in deep 3D imaging and confirmed the high accuracy of our method for label-free nucleolus identification using co-registered immunostaining and electron microscopy. To establish the biological relevance of our approach, we demonstrate that nucleolar stress leads to an unexpected depletion of NADH across cellular compartments. Furthermore, in the human endometrium—where nucleolar dynamics are central to the tissue’s response to progesterone—our label-free imaging strategy delineated endometrial structures in freshly excised tissues and revealed that progesterone treatment induces distinct changes in nucleolar translocation and metabolic adaptation in organoids derived from diseased patients compared to controls. This capacity to non-invasively visualize and quantify features of the nucleolus and its local metabolic microenvironment at single-cell resolution in human tissues—and dynamically track these changes over time in patient-derived organoids—provides a powerful tool for uncovering the roles of the nucleolus in development, disease progression, and therapeutic response. Together, these findings establish our platform as a significant advance for both fundamental research and organelle-based tissue diagnostics.

## Full Text Availability

The license terms selected by the author(s) for this preprint version do not permit archiving in PMC. The full text is available from the preprint server.

